# Supplementary material for: Causal interplay between lactose intolerance and gut microbiota: a combined bidirectional Mendelian randomization and in vivo validation study
Source: Front Nutr. 2026 Jun 1;13:1803337. doi: 10.3389/fnut.2026.1803337 (PMC13265576; doi:10.3389/fnut.2026.1803337)
Supplement: Supplementary file 1 [file Data_Sheet_1.zip › supplementary materials/Forward/forest plot/ebi-a-GCST90027637.finngen_R12_E4_LACTONAS.pdf]

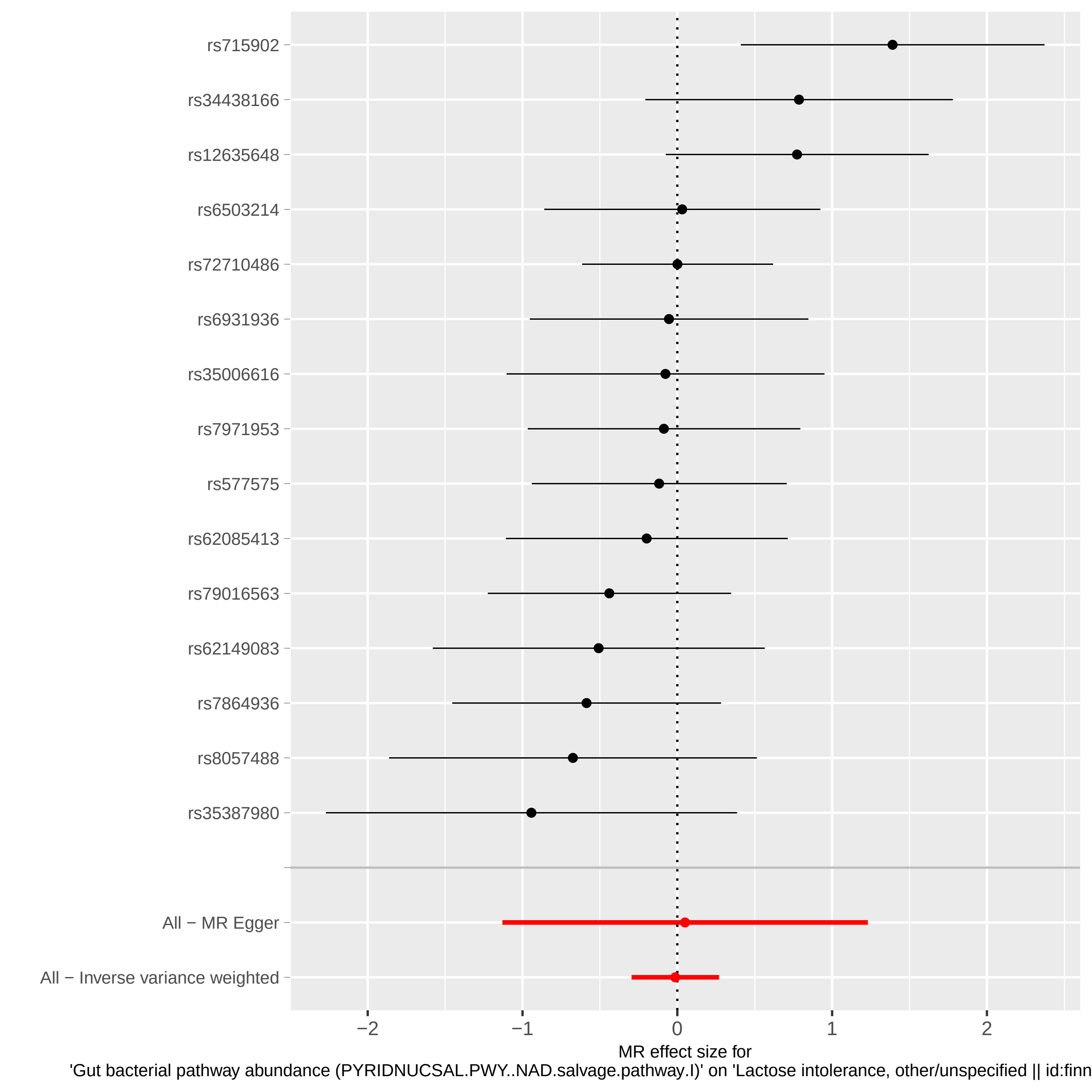
rs715902
rs34438166
rs12635648
rs6503214
rs72710486
rs6931936
rs35006616
rs7971953
rs577575
rs62085413
rs79016563
rs62149083
rs7864936
rs8057488
rs35387980
All − MR Egger
All − Inverse variance weighted
−2
−1
0
1
2
MR effect size for
'Gut bacterial pathway abundance (PYRIDNUCSAL.PWY..NAD.salvage.pathway.I)' on 'Lactose intolerance, other/unspecified || id:finn
